# Phase Structure and Mechanical Properties of Epoxy Resin Modified with Hydroxyl-Terminated Poly(methylphenylsiloxane)

**DOI:** 10.3390/polym18131569

**Published:** 2026-06-24

**Authors:** Xixuan He, Yundong Ji, Yu Zhao, Zhenxiang Guan, Dongfeng Cao, Zhentao Luo, Shuxin Li

**Affiliations:** 1 School of Materials Science and Engineering, Wuhan University of Technology, Wuhan 430070, China; 13871198069@163.com (X.H.); jiyundong@whut.edu.cn (Y.J.); 15135955297@163.com (Y.Z.); 2 China Railway 19 Bureau Group Co., Ltd., Beijing 100176, China; guanzhenxiang12@163.com; 3 State Key Laboratory of Advanced Technology for Materials Synthesis and Processing, Wuhan University of Technology, Wuhan 430070, China; shuxinwhut@whut.edu.cn; 4 Aerospace Science and Industry Wuhan Magnetism-Electron Co., Ltd., Wuhan 430070, China; luozhentaott@163.com; 5 Foshan Xianhu Laboratory of the Advanced Energy Science and Technology Guangdong Laboratory, Foshan 528000, China

**Keywords:** polysiloxane, epoxy resin, mechanical properties, phase structure

## Abstract

Bisphenol A type epoxy resin has the problem of relatively high brittleness after curing. Although traditional polysiloxane toughening methods can improve toughness, they often come at the expense of strength. In this paper, methylphenyl dimethoxysilane (MPS) was used as a monomer to synthesize end-hydroxyl poly(methylphenyl)siloxane (PMPS), which was then used to modify E51 epoxy resin. The structure and reaction degree were characterized by infrared spectroscopy, proton nuclear magnetic resonance spectroscopy, matrix-assisted laser desorption/ionization time-of-flight/time-of-flight mass spectrometry and viscosity tests. The mechanical test results show that when the PMPS content is 20 wt%, the tensile, flexural, compressive and impact strengths of the modified resin increase by 31.26%, 26.16%, 18.53% and 98.66%, respectively, compared with the unmodified resin, and the tensile and flexural elastic moduli increase by 38.36% and 32.25%, respectively. The fracture toughness increases by 60.29%, indicating that the strength, stiffness and toughness of the material have all been improved. Dynamic mechanical analysis shows that the glass transition temperature and crosslinking density of the system gradually decrease with increasing PMPS content. Thermogravimetric analysis shows that the introduction of PMPS increases the char yield and decreases the maximum thermal decomposition rate, thereby enhancing the thermal stability of the system. Microscopic morphology analysis by optical microscopy, scanning electron microscopy and atomic force microscopy shows that the system has good compatibility, and the internal different modulus phases are distributed in a network-like manner, forming a uniform co-continuous or bicontinuous phase structure. This structure effectively promotes stress transfer and energy dissipation, alleviates local stress concentration, and thus comprehensively improves the mechanical properties of the resin system.

## 1. Introduction

Bisphenol A type epoxy resins have been widely used in aerospace, electronic packaging, and high-performance composites due to its excellent adhesion, electrical insulation, and chemical resistance. However, such resins usually exhibit high internal stress and pronounced brittleness after curing, which limits their application under complex loading conditions [1,2]. Therefore, improving the toughness of epoxy resins while maintaining their high strength has become an important research focus in this field.

The toughening modification methods for epoxy resins mainly include rubber toughening, thermoplastic resin toughening, nanofiller reinforcement, and hyperbranched polymer modification. Rubber toughening [3] and thermoplastic resin toughening [4,5] can significantly improve impact toughness, but they are often accompanied by a reduction in mechanical strength. Nanofillers [6] can enhance both the toughness and tensile strength of epoxy resins; however, issues such as poor dispersion and high cost remain challenging. Hyperbranched polymer toughening [7,8] can improve the overall mechanical properties, but the synthesis process is relatively complex and economically unfavorable. In addition, although the incorporation of flexible chain segments [9,10] can relieve internal stress and improve impact toughness, it is often achieved at the expense of strength and modulus. Therefore, how to improve toughness while maintaining or even enhancing material strength remains a key challenge in current research.

The backbone of polysiloxane consists of flexible Si–O–Si chains. The Si–O bond possesses a bond length of approximately 0.165 nm, a bond angle of about 142°, and a bond energy of approximately 422.5 kJ/mol, which is significantly higher than those of C–C bonds (348 kJ/mol) and C–O bonds (326 kJ/mol). Introducing Si–O–Si structures into epoxy resin networks is expected to reduce internal stress during curing and improve the toughness of the system [11,12]. However, the solubility parameter of epoxy resin is approximately 10.0, whereas that of polysiloxane is only 7.4, resulting in poor thermodynamic compatibility between the two components [13]. Consequently, reaction-induced phase separation easily occurs during curing. Excessive phase separation destroys the homogeneity of the system and weakens the interfacial bonding strength, thereby reducing the mechanical strength of the material. Xie et al. [14] modified epoxy resin (EP-51) using hydroxyl-terminated polydimethylsiloxane (HTPDMS), and the results showed that the tensile strength of the modified epoxy decreased with increasing HTPDMS content, while the elongation at break and impact strength increased. Chen et al. [12] used polyphenylpropylsiloxane (PPPS) to modify epoxy resin, and when the PPPS content reached 40 wt%, the elongation at break and notched impact strength increased significantly; however, the dispersed phase diameter reached 2.8 μm, and the tensile strength decreased to only 30% of that of the neat resin. Scanning electron microscopy (SEM) characterization revealed that both systems formed a typical “sea–island” morphology, in which the polysiloxane-rich phase was dispersed within the epoxy matrix. Although this structure is beneficial for crack deflection and energy dissipation, the weak interfacial bonding can easily lead to stress concentration and microcrack formation, which is the primary reason for the increase in toughness accompanied by a reduction in strength.

To address the above issues, improving the compatibility between the two phases is considered crucial for achieving the synergistic optimization of mechanical properties. Researchers have introduced reactive functional groups such as amino, epoxy, or hydroxyl groups into the side chains or terminal groups of polysiloxanes, enabling them to react with epoxy resins and thereby enhance interfacial interactions while suppressing macroscopic phase separation [15]. Terminal functionalization not only regulates the compatibility of the system but also significantly affects the evolution of phase structures, which further determines the mechanical properties of the modified system. Sun et al. [16] synthesized an amino-propyl-terminated polysiloxane copolymer (APDMS) through ring-opening polymerization and used it to modify epoxy resin, obtaining a DGEBA/APDMS epoxy system. The results showed that the tensile strength of the modified system was improved, and SEM observations demonstrated that the siloxane phase was uniformly dispersed within the resin matrix without obvious phase separation. Jia et al. [17] synthesized an epoxy-functionalized hyperbranched polysiloxane (HPSi) and introduced it into an epoxy/methylphenyl silicone resin (Si603) system as a compatibilizer. SEM results indicated that the compatibility of the modified system was significantly improved. Although the tensile strength slightly decreased, the overall variation was relatively small. In addition, Pan from our research group [18] modified AG80 epoxy resin using hydroxyl-terminated polyphenylpropylsiloxane (Z-6018) and introduced a self-synthesized epoxy compatibilizer (P/E30) to regulate the resin phase structure. The results showed that the tensile strength and impact strength of the modified system increased by 50.89% and 454.79%, respectively, while SEM images revealed the formation of fine and uniformly dispersed siloxane phases within the system. Depending on the degree of copolymerization between polysiloxane and epoxy resin, the system may form either a sea–island structure or a co-continuous phase structure. Compared with the sea–island structure, the co-continuous structure is more favorable for stress transfer and energy dissipation, thereby enabling the simultaneous enhancement of strength and toughness. Therefore, regulating the degree of copolymerization to optimize the microphase structure is critical for improving the overall performance of epoxy resins.

In our group’s previous work, hydroxyl-terminated polyphenylpropylsiloxane (PPPS) was used to modify E51 epoxy resin, and a transition from a sea–island structure to a co-continuous phase structure was achieved by adjusting the additive content, resulting in the synergistic improvement of strength and toughness within a certain range. Studies demonstrated that the formation of a co-continuous structure is closely related to the degree of participation of polysiloxane in the curing reaction [19,20,21]. Generally, the degree of chemical copolymerization can be enhanced by introducing reactive functional groups, using suitable catalysts or coupling agents, or rationally designing the molecular structure. Increasing the copolymerization degree and adjusting the polysiloxane content to suppress phase separation and promote the formation of a co-continuous phase are key strategies for epoxy resin modification. Based on the above studies, this work proposes suppressing phase separation and promoting the formation of a co-continuous structure by regulating the polysiloxane content, thereby achieving the synergistic enhancement of toughness and strength. Compared with PPPS, low-molecular-weight hydroxyl-terminated polymethylphenylsiloxane (PMPS) possesses a higher hydroxyl content per unit mass, while the methyl side groups can reduce steric hindrance and improve reaction activity, facilitating its incorporation into the epoxy curing network. Therefore, PMPS is expected to exhibit phase structure evolution behavior and property regulation characteristics different from those of PPPS. It is anticipated that rational control of PMPS content can effectively optimize the phase structure of the system and simultaneously improve its mechanical properties.

In this study, hydroxyl-terminated polymethylphenylsiloxane was synthesized using methylphenyldimethoxysilane as the monomer and subsequently applied to modify E51 epoxy resin. The structure of the synthesized product was characterized by Fourier transform infrared spectroscopy (FTIR), proton nuclear magnetic resonance (^1^H NMR) and matrix-assisted laser desorption/ionization time-of-flight/time-of-flight mass spectrometry (MALDI-TOF/TOF MS). The degree of copolymerization was evaluated through epoxy value determination, viscosity measurements, and optical microscopy observations of copolymer morphologies. Furthermore, the phase structure characteristics of the system were analyzed using SEM and atomic force microscopy (AFM). On this basis, dynamic mechanical analysis (DMA) was employed to characterize the dynamic thermomechanical behavior of the materials and to assist in evaluating the effects of crosslinking density and phase structure evolution on material properties through storage modulus and glass transition behavior. Meanwhile, thermogravimetric analysis (TG/DTG) was conducted to evaluate the thermal stability of the materials, providing supplementary evidence for elucidating the relationship between structure and properties. Combined with mechanical performance testing, the influence of phase structure evolution on the properties of epoxy resins was systematically investigated.

## 2. Materials and Methods

### 2.1. Materials

The materials used in this study include: Epoxy Resin (EP, epoxy value of 0.51, industrial grade) was purchased from Sinopec Hunan Petrochemical Co., Ltd. (Yueyang, China). Methylphenyldimethoxysilane (MPS, chemically pure) was purchased from J&K Scientific Ltd. (Beijing, China). Dibutyltin Dilaurate (DBDTL, industrial grade) and 4-Methylhexahydrophthalic Anhydride (MHHPA, industrial grade) were purchased from Shandong Keyuan Biochemical Co., Ltd. (Laizhou, China). 2,4,6-Tris(dimethylaminomethyl)phenol (DMP-30, analytical grade) and Acetone (analytical grade) were purchased from Sinopharm Chemical Reagent Co., Ltd. (Shanghai, China).

### 2.2. Synthesis of Hydroxyl-Terminated Polymethylphenylsiloxane

**Figure 1:**
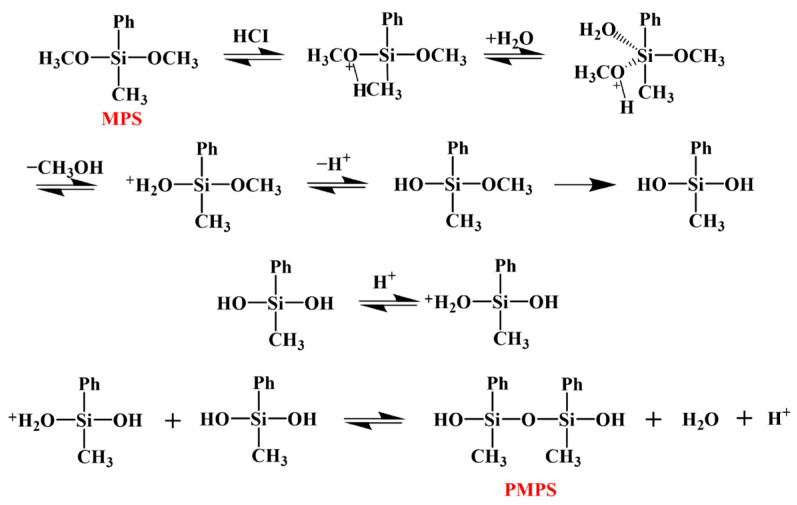
Schematic illustration of the PMPS synthesis route.

Figure 1 shows the synthetic route of PMPS. A 250 mL three-neck flask equipped with a mechanical stirrer, a reflux condenser, and a temperature probe was used as the reaction vessel. Subsequently, 25 mL of Methylphenyldimethoxysilane (MPS) and 50 mL of deionized water were added into the flask. The reaction system was placed in a water bath and heated to 75 °C under a stirring speed of 250 rpm, followed by the addition of 1.5 mL of concentrated hydrochloric acid. The reaction was maintained under these conditions for 6 h. After completion of the reaction, the oil phase was washed 5–6 times with deionized water. The pH value of the product was monitored using pH test paper, and the washing process was considered complete when the pH reached approximately 7. The lower oily translucent liquid was separated using a separatory funnel and dried in a vacuum oven for 8 h, yielding a colorless, transparent, viscous liquid product, namely hydroxyl-terminated polymethylphenylsiloxane (PMPS).

### 2.3. Preparation of PMPS-Modified Epoxy Resin

**Figure 2:**
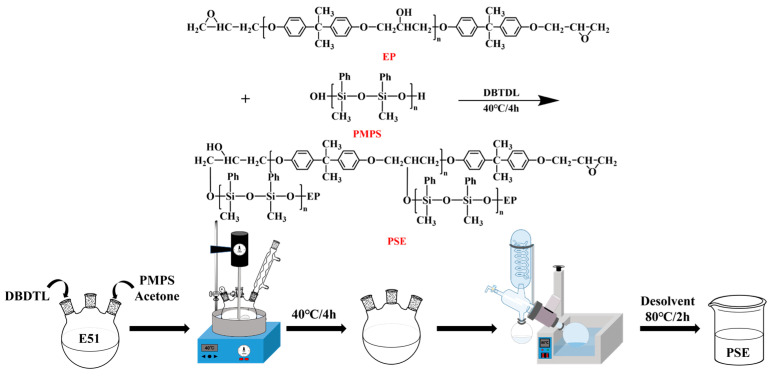
Schematic illustration of the PSE synthesis route.

Figure 2 shows the synthetic route of PSE. A 250 mL three-neck flask equipped with a mechanical stirrer, reflux condenser, and temperature probe was used as the reaction vessel. PMPS and Epoxy Resin (E51) were added according to mass ratios of 1:9, 2:8, 3:7, and 4:6, respectively. Subsequently, acetone with the same mass as the epoxy resin was added as the solvent. The reaction system was heated to 40 °C in a water bath under a stirring speed of 250 rpm, followed by the addition of Dibutyltin Dilaurate (DBTDL) at 1.5 wt% of the total mass of PMPS and E51. The reaction was carried out at 40 °C and 250 rpm for 4 h. The obtained colorless transparent liquid was transferred into a rotary evaporation flask and rotary-evaporated at 80 °C for 2 h to remove the solvent, ultimately yielding a colorless transparent viscous liquid product. The samples were designated according to the mass fraction of PMPS in the epoxy resin system, such as PSE10, where “10” represents the percentage of PMPS in the total resin content. The samples were labeled as PSE0 (pure E51 epoxy resin), PSE10, PSE20, PSE30, and PSE40, respectively.

### 2.4. Preparation of Cured PSE Resins

A certain amount of PSE resin was weighed, followed by the calculated addition of the curing agent MHHPA according to the epoxy value of the system, and the accelerator DMP-30 was added based on the resin weight. The detailed formulations are listed in Table 1. The mixture was thoroughly stirred until homogeneous. The resulting mixture was placed in a vacuum oven at 60 °C and degassed under a vacuum of 0.1 MPa for 20 min until all bubbles were completely removed. The molds were uniformly coated twice with a release agent and preheated in an oven at 80 °C for 30 min. The degassed resin mixture was then slowly poured along the edge into the mold for curing. The curing procedure was conducted as follows: 100 °C/2 h + 120 °C/1 h + 150 °C/5 h. After cooling to room temperature, the cured resin sheets were obtained.

### 2.5. Characterization Methods

#### 2.5.1. Fourier Transform Infrared Spectroscopy

The chemical compositions of the samples were analyzed using a Fourier transform infrared spectrometer (Nicolet 6700, Thermo Fisher Scientific, Waltham, MA, USA). The spectra were recorded in the range of 4000–400 cm^−1^.

#### 2.5.2. Proton Nuclear Magnetic Resonance

The molecular structures of the samples were characterized using a nuclear magnetic resonance spectrometer (AV400, KREIENBAUM Neoscience GmbH, Langenfeld, Germany). The samples were dissolved in CDCl_3_ for ^1^H NMR measurements.

#### 2.5.3. Matrix-Assisted Laser Desorption/Ionization Time-of-Flight/Time-of-Flight Mass Spectrometry

The molecular weight and structure of PMPS were characterized using matrix-assisted laser desorption/ionization time-of-flight/time-of-flight mass spectrometry (MALDI-TOF/TOF MS, Bruker Daltonics, Bremen, Germany). 2,5-Dihydroxybenzoic acid was used as the matrix.

#### 2.5.4. Optical Microscopy

An optical microscope (MSD-S750, Maishidi (Dongguan) Technology Co., Ltd., Dongguan City, China) was employed to observe the compatibility and phase morphology of the PSE systems.

#### 2.5.5. Determination of Epoxy Value of PSE Resins

The epoxy value of the PSE resins was determined according to the GB/T 1677–2023 standard [22] using the hydrochloric acid–acetone method. A 0.5 g resin sample (accurate to 0.001 g) was weighed into a 250 mL conical flask. Subsequently, 20 mL of hydrochloric acid–acetone solution was measured using a graduated cylinder and added to dissolve the sample. After thorough mixing, the flask was placed in the dark and allowed to stand for 30 min. Phenolphthalein was used as the indicator, and the sample solution was titrated with the prepared sodium hydroxide standard solution until a pale pink color appeared and remained unchanged for 30 s. The volume of sodium hydroxide solution consumed was recorded. A blank test was conducted following the same procedure.

The epoxy value was calculated according to the following equation:

X_1_ = 100 × [V − (V_1_ − V_2_/G × W)] × N × 0.016/W (1)

V—volume of sodium hydroxide standard solution consumed in the blank test (mL);
V_1_—volume of sodium hydroxide standard solution consumed in the sample test (mL);
V_2_—volume of sodium hydroxide standard solution consumed in the acid value determination (mL);
N—concentration of the sodium hydroxide standard solution (mol/L);
W—mass of the sample used for epoxy value determination (g);
G—mass of the sample used for acid value determination (g);
0.016—molar mass of oxygen (g/mol).

#### 2.5.6. Viscosity Measurement

The viscosities of the PSE resins were measured using a digital rotational viscometer (DNJ-8S, Shanghai Fangrui Instrument Co., Ltd., Shanghai City, China) at an ambient temperature of 25 °C.

#### 2.5.7. Mechanical Property Testing

The mechanical properties of the cured PSE resins were tested according to GB/T 2567-2021 standards [23], while the fracture toughness of the cured PSE resins was determined according to GB/T 41932-2022 [24]. Tensile, flexural, and compression tests were performed using a universal testing machine (Instron 5967, Instron, Norwood, MA, USA), while impact performance was evaluated using an impact testing machine (XJJ-50, Donglai Instrument Testing Co., Ltd., Chengde City, China). Fracture toughness tests were also performed using the universal testing machine. The tensile specimens had dimensions of 200 mm × (10 ± 0.2) mm × (4.0 ± 0.2) mm. The flexural specimens had dimensions of 80 mm × 15 mm × (4.0 ± 0.2) mm. The compression and impact specimens had dimensions of 55 mm × (10 ± 0.2) mm × (10 ± 0.2) mm. The fracture toughness (K_IC_) specimens had dimensions of 40 mm × 8 mm × 4 mm, with a crack length of 3.6–4.4 mm.

For tensile testing, a static tensile load was uniformly applied along the axial direction of the specimen at a constant crosshead speed until fracture, and the tensile strength and tensile modulus were determined. For flexural testing, a constant loading rate was applied through the upper loading head until specimen failure, and the flexural strength and flexural modulus were measured. Compression tests were conducted by applying a compressive load along the axial direction at a constant rate until specimen failure or a predetermined deformation was reached, and the compressive strength was recorded. For impact testing, the pendulum was raised and locked, and the specimen was placed tightly on the support with the impact center aligned to the center of the specimen. The pendulum was then released steadily to determine the impact strength. For fracture toughness testing, a three-point bending configuration equipped with a movable roller fixture was employed. The specimen was loaded at a constant rate until fracture, and the fracture toughness was determined.

All tests were conducted at room temperature. All mechanical property results were averaged from five independent specimens.

#### 2.5.8. Morphological Characterization

The fracture surface morphologies of the samples were observed using a scanning electron microscope (Zeiss Ultra Plus, ZEISS, White Plains, NY, USA) under an accelerating voltage of 10 kV. Prior to testing, impurities on the sample surfaces were carefully removed, and the fracture surfaces were sputter-coated with gold to improve electrical conductivity.

An atomic force microscope (Bruker Dimension ICON, Bruker, Billerica, MA, USA) was operated in PeakForce tapping mode to characterize the surface morphology and modulus distribution of the samples.

#### 2.5.9. Dynamic Mechanical Analysis

Dynamic mechanical analysis was conducted using a dynamic mechanical analyzer (DMA 8000, TA Instruments, New Castle, DE, USA). The specimen dimensions were 35 mm × 10 mm × 2 mm. Measurements were performed in three-point bending mode at a frequency of 1 Hz. The temperature was increased from 30 °C to 280 °C at a heating rate of 5 °C/min.

#### 2.5.10. Thermogravimetric Analysis

Thermogravimetric analysis was carried out using a simultaneous thermal analyzer (STA449F3, PerkinElmer, Rodgau, Germany). The cured resin samples were heated from room temperature to 1000 °C under air and nitrogen atmospheres at a heating rate of 10 °C/min.

## 3. Results and Discussion

### 3.1. Structural Characterization of PSE Resins

**Figure 3:**
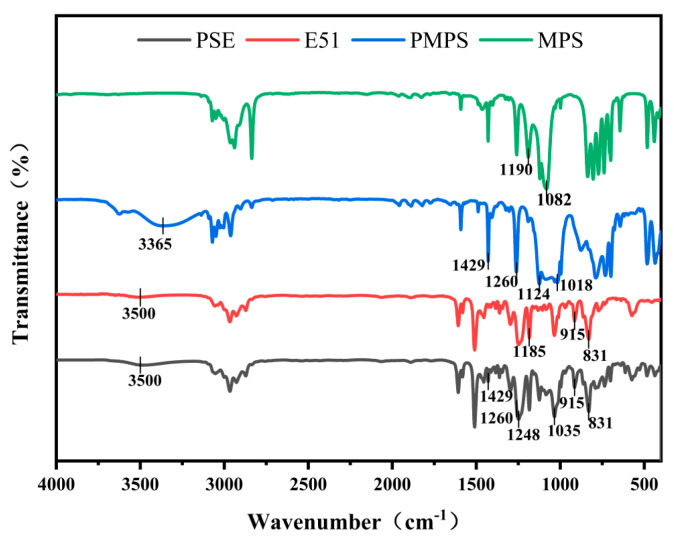
FTIR spectra of MPS, PMPS, E51, and PSE.

Figure 3 presents the FTIR spectra of MPS, PMPS, E51, and PSE. By comparing the spectra of MPS before and after hydrolysis, a new absorption peak corresponding to silanol groups appeared at 3365 cm^−1^ in the PMPS spectrum. The characteristic absorption peak of the Si–O–CH_3_ bond observed at 1190 cm^−1^ in MPS disappeared after hydrolysis. In addition, MPS exhibited a single stretching vibration peak of the Si–O bond at 1082 cm^−1^, whereas the broad absorption band appearing in the range of 1018–1124 cm^−1^ in PMPS corresponded to the characteristic stretching vibration of Si–O–Si bonds. These results indicate that the Si–O bonds in MPS underwent hydrolysis–condensation reactions to form Si–O–Si linkages. Based on the above analysis, the synthesized product can be preliminarily identified as hydroxyl-terminated polymethylphenylsiloxane (PMPS) [12,25,26].

Comparisons among the FTIR spectra of PMPS, E51, and PSE further confirmed the successful modification of E51 by PMPS. In the PMPS spectrum, the characteristic absorption peaks located at 1018–1124 cm^−1^, 1429 cm^−1^, and 1260 cm^−1^ corresponded to Si–O–Si, Si-Ph, and Si–CH_3_ groups, respectively. In the E51 spectrum, the absorption peak at 3500 cm^−1^ was assigned to hydroxyl groups, the peak at 1185 cm^−1^ corresponded to C–O–C bonds, and the peaks at 915 cm^−1^ and 831 cm^−1^ were attributed to the stretching and bending vibrations of epoxy groups. Compared with neat E51, new characteristic absorption peaks appeared at 1429 cm^−1^ and 1260 cm^−1^ in the PSE spectrum, corresponding to silicon-bonded phenyl and methyl groups, while the remaining absorption peaks were generally consistent with those of E51. These results suggest that PMPS was incorporated into the epoxy resin system. Furthermore, the hydroxyl absorption peak at 3500 cm^−1^ became slightly stronger due to the introduction of terminal hydroxyl groups from PMPS and the ring-opening reaction between PMPS hydroxyl groups and epoxy terminal groups. Since the characteristic absorption peaks of Si–O–C bonds overlapped with those of Si–O–Si (1018–1124 cm^−1^) and C–O–C (1185 cm^−1^), a broad absorption band was observed in the range of 1035–1248 cm^−1^. Meanwhile, the epoxy group absorption peaks at 915 cm^−1^ and 831 cm^−1^ weakened in the PSE spectrum, indicating that the hydroxyl groups in PMPS reacted with the epoxy groups in E51 through ring-opening reactions to form crosslinked structures. Based on the above results, PMPS was successfully introduced into the epoxy resin system and participated in the modification process.

**Figure 4:**
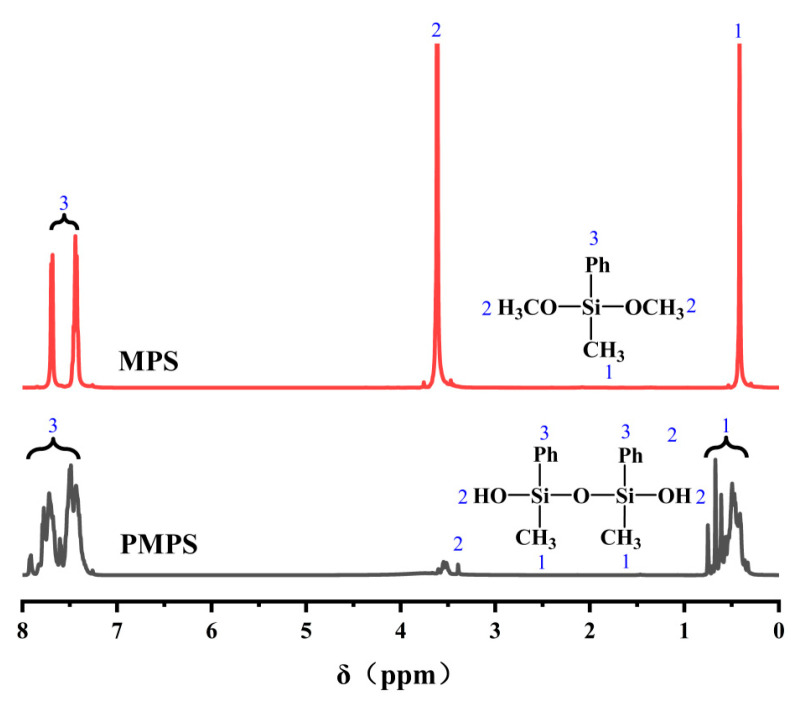
^1^H NMR spectra of MPS and PMPS.

Figure 4 compares the ^1^H NMR spectra of MPS and PMPS. The characteristic chemical shift peaks of MPS were observed at 0.4 ppm (Si–CH_3_), 3.6 ppm (–OCH_3_), and 7.5–7.7 ppm (benzene ring). In contrast, the characteristic peaks of PMPS appeared at 0.5 ppm (Si–CH_3_), 3.4 ppm (Si–OH), and 7.4–7.8 ppm (benzene ring). Due to the incomplete hydrolysis of some methoxy groups, a weak residual peak corresponding to –OCH_3_ was still observed at 3.6 ppm. These results further confirm that the synthesized product was PMPS.

**Figure 5:**
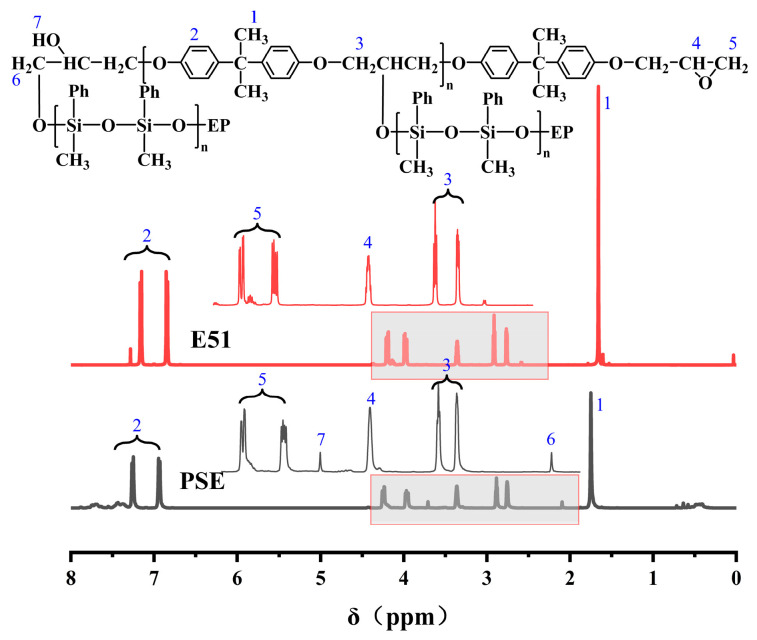
^1^H NMR spectra of E51 and PSE.

Figure 5 compares the ^1^H NMR spectra of E51 and PSE. The characteristic chemical shift peaks of E51 included 1.7 ppm (–CH_3_), 2.7–2.9 ppm (–CH_2_–), 3.3 ppm (–CH–), 3.9–4.2 ppm (–O–CH_2_–), and 6.8–7.2 ppm (benzene ring). In the PSE spectrum, new chemical shift peaks appeared at 2.1 ppm (–CH_2_–O–Si) and 3.7 ppm (–CH(OH)–), which were attributed to the ring-opening reaction between epoxy terminal groups and hydroxyl groups. The relatively low peak intensity suggests that the reaction degree was limited, which is consistent with the low PMPS content. In addition, weak absorption peaks at 0.4–0.6 ppm corresponding to Si–CH_3_ groups indicated the presence of a small amount of unreacted PMPS. These findings provide further evidence that PMPS participated in the modification process of the epoxy resin.

**Figure 6:**
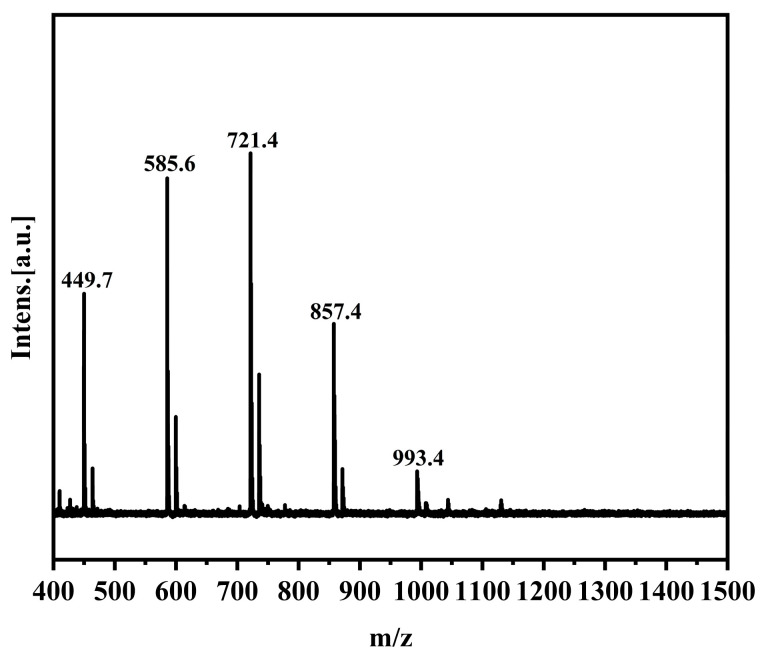
MALDI-TOF/TOF MS spectrum of PMPS.

Figure 6 shows the MALDI-TOF/TOF MS spectrum of PMPS. A series of molecular ion peaks were observed at *m*/*z* 449.7, 585.6, 721.4, 857.4, and 993.4. The constant mass difference of 136 Da between adjacent peaks corresponds to the molecular weight of the repeating unit of PMPS, confirming the expected oligomeric structure and the proposed polymerization mechanism. During MALDI analysis, polymer molecules are commonly ionized as alkali metal cation adducts, with sodium adducts ([M + Na]^+^) being the predominant species [27]. Consequently, the observed *m*/*z* values are 23 Da higher than the corresponding neutral molecular weights, giving calculated molecular weights of 426, 562, 698, 834, and 970, respectively. The assigned molecular structures and the corresponding numbers of repeating units for each peak are summarized in Table 2. These results indicate that the synthesized PMPS mainly consists of linear oligomers, and no cyclic by-products were detected.

### 3.2. Optical Microscopy Analysis of PSE Resins

**Figure 7:**
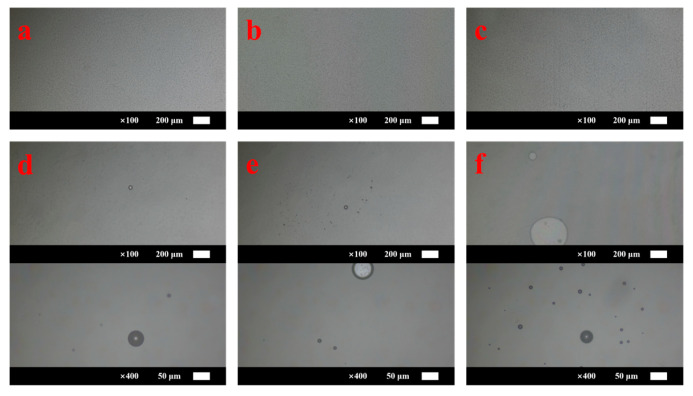
Optical microscopy images of modified epoxy resins: (**a**) neat epoxy resin, (**b**) PSE10, (**c**) PSE20, (**d**) PSE30, (**e**) PSE40, and (**f**) physical blend.

Figure 7 presents the optical micrographs of the PSE systems. The neat epoxy resin (Figure 7a) exhibited a uniform morphology. At low PMPS contents (Figure 7b,c), the systems remained transparent and homogeneous, with almost no dispersed droplets observed, indicating good compatibility between PMPS and E51. As the PMPS content increased (Figure 7d,e), a small number of fine spherical structures gradually appeared within the matrix. Some dispersed phases could be observed under low magnification (100×); therefore, local regions were further examined under higher magnification (400×). The results showed that these spherical structures were relatively small and uniformly dispersed, with most sizes remaining within the micrometer range and without obvious large-scale aggregation, suggesting that no severe macroscopic phase separation occurred in the system. Since air may have been introduced during sample preparation, some spherical structures were likely residual microbubbles rather than phase-separated droplets. In contrast, the physically blended system (Figure 7f) exhibited significantly larger and more numerous PMPS droplets, indicating much poorer compatibility than the chemically modified systems. Overall, although the compatibility slightly decreased at high PMPS contents, the chemically modified PSE systems still maintained good miscibility and predominantly homogeneous morphologies.

### 3.3. Determination of Epoxy Value of PSE Resins

**Figure 8:**
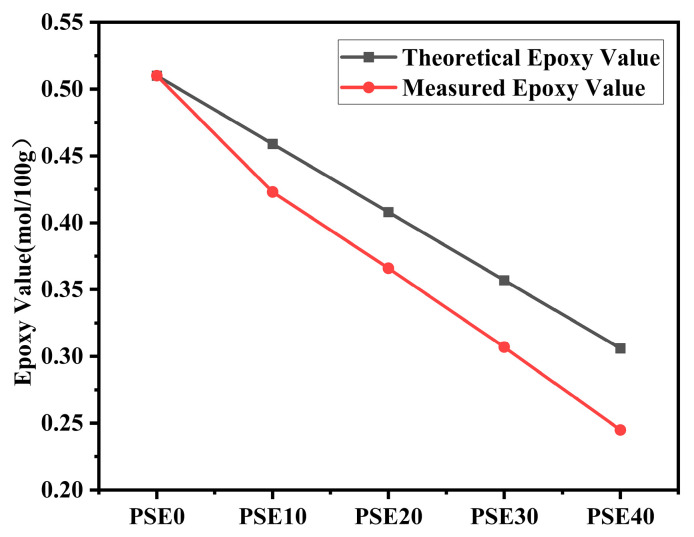
Theoretical and measured epoxy values of PSE resins.

Figure 8 presents the theoretical and measured epoxy values of the PSE systems. The theoretical epoxy value was calculated based on the epoxy value of E51 and its mass fraction in the system. As the PMPS content increased, the theoretical epoxy value gradually decreased due to the reduced proportion of epoxy resin in the formulation. The measured epoxy values of all samples were lower than the corresponding theoretical values, and the epoxy group consumption rate increased from 7.64% for PSE10 to 19.67% for PSE40, indicating that the conversion degree of epoxy groups gradually increased with increasing PMPS content. This behavior is likely associated with the ring-opening reaction between the Si–OH groups in PMPS and the epoxy groups in E51. As the PMPS content increased, more hydroxyl groups became available to participate in the reaction, thereby promoting the continuous consumption of epoxy groups. Combined with the FTIR and ^1^H NMR results, these findings provide further evidence that PMPS participated in the modification process of the epoxy resin. Since the overall consumption of epoxy groups was limited, both chemically grafted structures and unreacted components coexisted in the system.

### 3.4. Viscosity Analysis of PSE Resins

**Figure 9:**
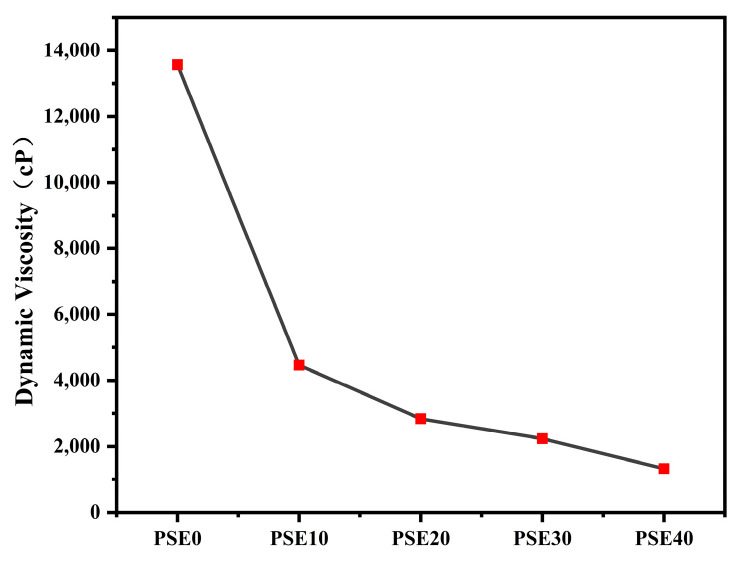
Variation in viscosity of PSE samples.

As shown in Figure 9, the dynamic viscosity of the PSE systems decreased significantly with increasing PMPS content. The dynamic viscosity of neat epoxy resin was 13,563.42 cP. After the introduction of PMPS, the viscosity of the system decreased rapidly. The largest reduction occurred in the low-content range (PSE0–PSE10), where the viscosity decreased by approximately 70%, after which the decreasing trend gradually slowed. This behavior can be attributed to the flexible Si–O–Si backbone of PMPS, which possesses low cohesive energy and high chain mobility. The incorporation of PMPS weakens intermolecular interactions among epoxy chains and reduces chain entanglement, thereby decreasing the flow resistance of the system [28]. As the PMPS content further increased, the system already exhibited relatively low viscosity, and the viscosity-reducing effect of PMPS gradually approached saturation.

It is noteworthy that the viscosity decrease became less pronounced in the PSE20–PSE30 range, whereas the decreasing trend slightly intensified from PSE30 to PSE40, indicating a nonlinear viscosity evolution behavior. At intermediate PMPS contents, the epoxy molecular chains had already been sufficiently plasticized, and further reduction of chain entanglement became limited. At higher PMPS contents, however, the siloxane segments gradually dominated the flow behavior of the system, which became increasingly governed by low-viscosity components. This may lead to the formation of continuous low-resistance flow pathways, resulting in another significant decrease in viscosity [19].

### 3.5. Mechanical Properties and Phase Morphology Analysis of PSE Resins

#### 3.5.1. AFM Phase Structure Analysis of PSE

**Figure 10:**
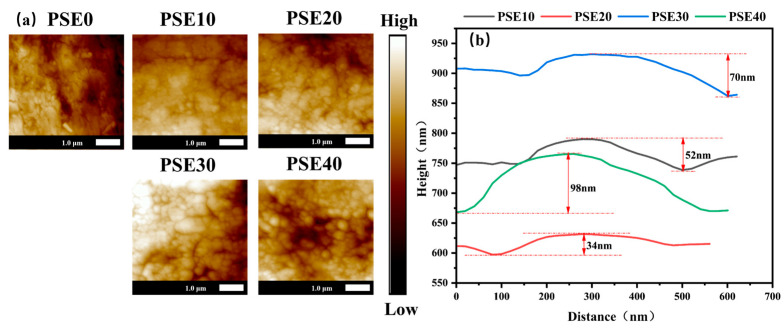
AFM characterization of the PSE systems: (**a**) Height Sensor images of the PSE systems. (**b**) Phase height variation profiles of the PSE systems.

The phase structures of the PSE systems were characterized by AFM, and the results are shown in Figure 10. As shown in Figure 10a, the surface of the cured neat epoxy resin (PSE0) was relatively smooth, indicating a homogeneous internal structure. After the introduction of PMPS, raised phase domains gradually appeared on the cured resin surface. In the PSE10 and PSE20 systems, the phase domains were relatively small in size and limited in number, resulting in weak surface undulations, which indicates good compatibility between PMPS and E51. Quantitative analysis of the height variation in the PMPS phase in the AFM images (Figure 10b) showed that the height differences between the phase domains and the matrix were approximately 52 nm and 34 nm, respectively. When the PMPS content increased to 30 wt% and 40 wt%, the raised phase domains became more numerous and gradually interconnected, accompanied by a significant increase in domain size and surface roughness. The corresponding height differences increased to approximately 70 nm and 98 nm, respectively. These results indicate that the size of the phase domains and the morphological differences within the system gradually increased with increasing PMPS content.

**Figure 11:**
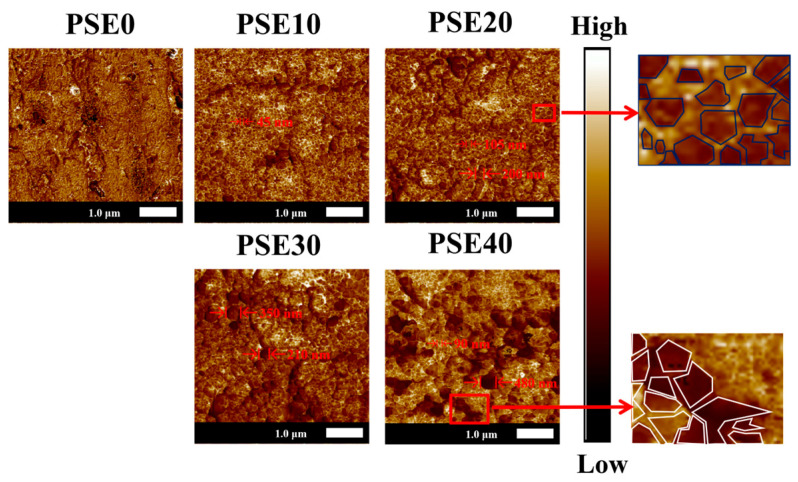
AFM-DMT modulus images of PSE systems.

Figure 11 shows the DMT modulus images of the PSE systems. The neat epoxy resin exhibited a uniform modulus distribution, characteristic of a homogeneous crosslinked network structure. After introducing small amounts of PMPS (PSE10–PSE20), distinct low-modulus soft domains appeared. These regions possessed nanoscale dimensions and exhibited finely dispersed alternating bright/dark (high-/low-modulus) distributions. This indicates that although PMPS and epoxy resin began to phase-separate, the presence of terminal hydroxyl groups enabled chemical interactions between the two phases, resulting in good interfacial compatibility and the formation of nanoscale co-continuous structures. When the PMPS content increased to PSE30–PSE40, the system still maintained a network-like morphology; however, the modulus contrast between phases became more pronounced and the distribution became less uniform. Large low-modulus dark domains with weakened connectivity were observed, suggesting a decrease in compatibility. This phenomenon can be attributed to the excessive incorporation of flexible PMPS chains at high contents, which reduced the crosslink density and diluted the network structure, thereby weakening interfacial constraints.

**Figure 12:**
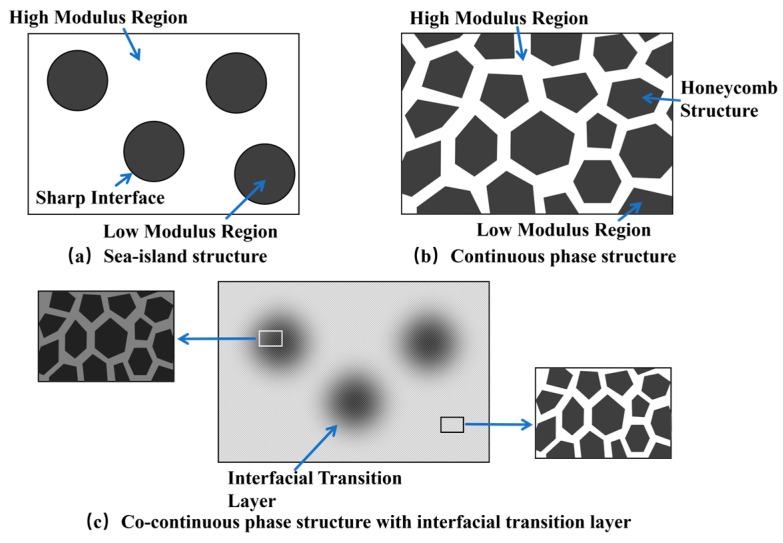
Schematic illustration of the co-continuous phase structure with interfacial transition layers.

Notably, unlike conventional “sea–island” morphologies with clear particle–matrix boundaries, the PSE systems did not exhibit distinct spherical phase boundaries even at relatively high PMPS contents. AFM results revealed that the PSE40 system exhibited relatively obvious soft–hard phase separation characteristics; however, the high- and low-modulus regions still maintained a certain degree of connectivity and displayed mutually interconnected network-like distributions. As illustrated in Figure 12, the soft and hard phases were not simply dispersed independently but instead formed co-continuous morphologies with a certain degree of continuity. This behavior may be associated with the interactions between PMPS and the epoxy resin matrix, which could improve the continuity of the two phases and contribute to the observed interconnected morphology. Although increasing PMPS content intensified phase separation, the interconnected morphology may alleviate abrupt modulus differences between neighboring regions compared with conventional phase-separated systems, producing a more gradual mechanical transition between the soft and hard phases while maintaining structural continuity between the two phases. Overall, the incorporation of PMPS promoted the formation of co-continuous network structures in the PSE systems, effectively suppressing severe interfacial defects and local stress concentrations commonly observed in phase-separated systems. Moreover, this structure facilitated uniform stress transfer and reduced crack propagation rates through mechanisms such as crack deflection within the flexible PMPS phase, thereby achieving the synergistic enhancement of strength, stiffness, and toughness.

**Figure 13:**
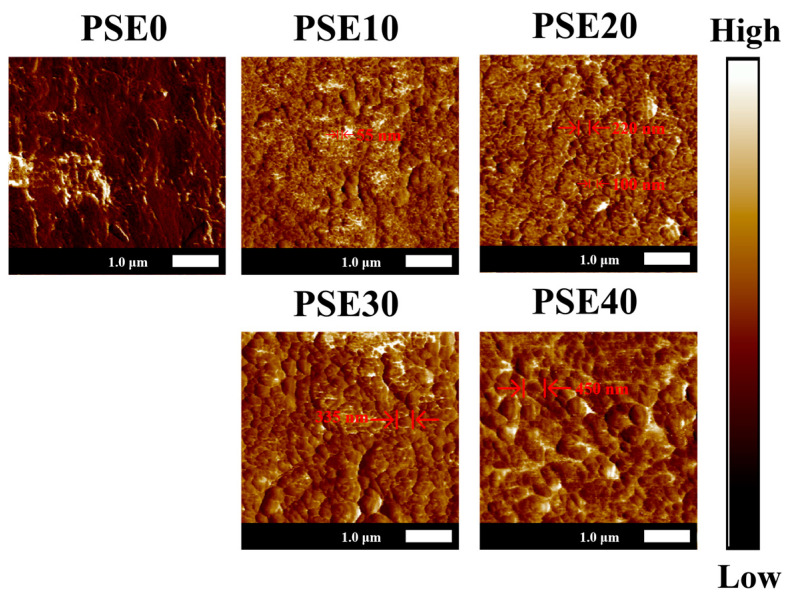
AFM adhesion images of PSE systems.

AFM adhesion images, which are based on the spatial distribution of probe pull-off forces, can effectively characterize differences in interfacial interactions among different phases, thereby revealing phase distribution, phase size, and interfacial compatibility evolution in multiphase systems [29]. Figure 13 presents the adhesion images of the PSE systems, further illustrating the phase evolution process. The neat epoxy resin (PSE0) exhibited relatively uniform adhesion distribution, indicating a homogeneous crosslinked network. With the incorporation of PMPS (PSE10–PSE20), low-adhesion regions corresponding to PMPS-rich soft phases gradually appeared. These regions were uniformly distributed and finely dispersed, indicating the formation of nanoscale co-continuous structures with good interfacial compatibility. At higher PMPS contents (PSE30–PSE40), the low-adhesion regions became larger and more aggregated, and the distribution became less uniform, suggesting increased phase separation and reduced interfacial compatibility. Nevertheless, the phase domains still remained within the micrometer scale without obvious macroscopic phase separation. Notably, the spatial distribution of adhesion contrast was consistent with that observed in the DMT modulus images. The changes in adhesion contrast indicate a gradual transition from uniformly dispersed co-continuous structures to coarsened morphologies with increasing PMPS content.

#### 3.5.2. Tensile Properties and Morphological Analysis of PSE Resins

**Figure 14:**
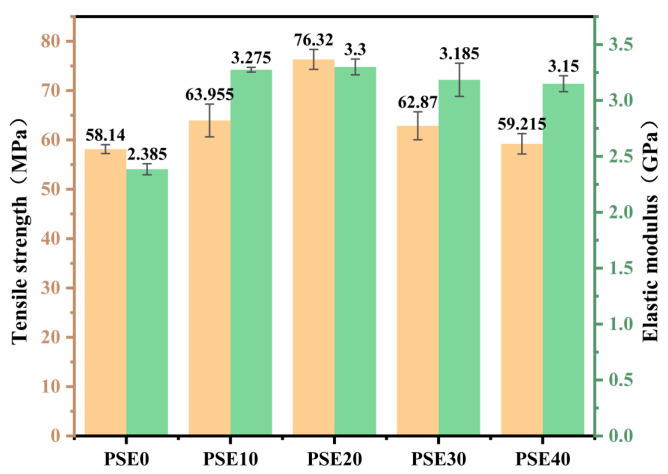
Tensile strength and tensile modulus of PSE samples.

Figure 14 presents the variations in tensile strength and tensile modulus of the PSE systems with increasing PMPS content. As the PMPS content increased, the tensile strength increased from 58.14 MPa for PSE0 to 76.32 MPa for PSE20, representing an improvement of 31.26% compared with the unmodified resin. Meanwhile, the tensile modulus increased from 2.385 GPa to 3.30 GPa, corresponding to an enhancement of 38.36%. However, both properties decreased at higher PMPS contents.

At low PMPS contents (≤20 wt%), the improvement in tensile properties can be attributed to the ring-opening reaction between the Si–OH groups in PMPS and the epoxy groups in E51, which promoted interactions between PMPS and the epoxy network and contributed to the formation of a relatively uniform and continuous phase structure. This may facilitate stress transfer between different regions of the material. In addition, the rigid phenyl side groups and steric hindrance restricted the mobility of the Si–O–Si chains to a certain extent, allowing the system to achieve the synergistic enhancement of stiffness and strength while maintaining adequate toughness. When the PMPS content further increased (>20 wt%), the size and continuity of the PMPS-rich domains increased, accompanied by a higher proportion of flexible Si–O–Si chains and a reduction in the crosslink density of the resin network. Consequently, the epoxy matrix network became disrupted and relaxed. Combined with the optical morphology observations showing microscale phase separation, the PMPS-rich domains weakened the interfacial bonding and induced stress concentration, resulting in the deterioration of tensile properties.

**Figure 15:**
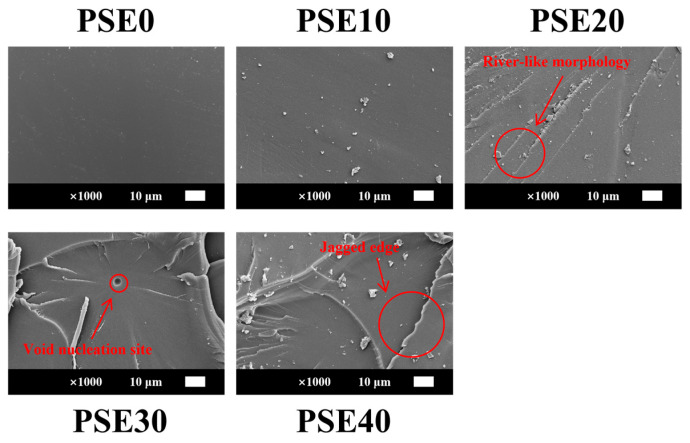
SEM images of tensile fracture surfaces of PSE resins.

The fracture morphologies shown in Figure 15 further support the above mechanisms. The neat epoxy resin (PSE0) exhibited a typical smooth and brittle fracture surface with limited energy dissipation capability [30]. After modification, the fracture surfaces gradually became rougher and were accompanied by crack deflection and branching, indicating a transition from brittle fracture to ductile fracture behavior. No obvious silicon-rich spherical domains were observed on the fracture surfaces, suggesting that the systems still maintained relatively homogeneous structures [1]. At low PMPS contents (PSE10 and PSE20), the fracture surfaces displayed river-like patterns and slight plastic deformation, suggesting that the crosslinked network remained relatively intact while the toughness was improved. The increased crack deflection and branching suggest that the flexible Si–O–Si chains facilitated energy dissipation and reduced stress concentration, thereby contributing to an optimal balance between strength and toughness. When the PMPS content increased to PSE30, obvious tear ridges and dimples containing central voids appeared, which may be associated with cavitation or interfacial debonding within the PMPS-rich regions during tensile deformation. These features are considered to promote induced plastic deformation in the surrounding matrix and enhanced energy absorption. However, at even higher PMPS content (PSE40), the fracture edges became rougher while the crack propagation resistance appeared to decrease, which may be attributed to further network relaxation and excessive softening. As a result, the load transfer efficiency decreased, leading to deterioration in the mechanical properties.

#### 3.5.3. Flexural Property Analysis of PSE Resins

**Figure 16:**
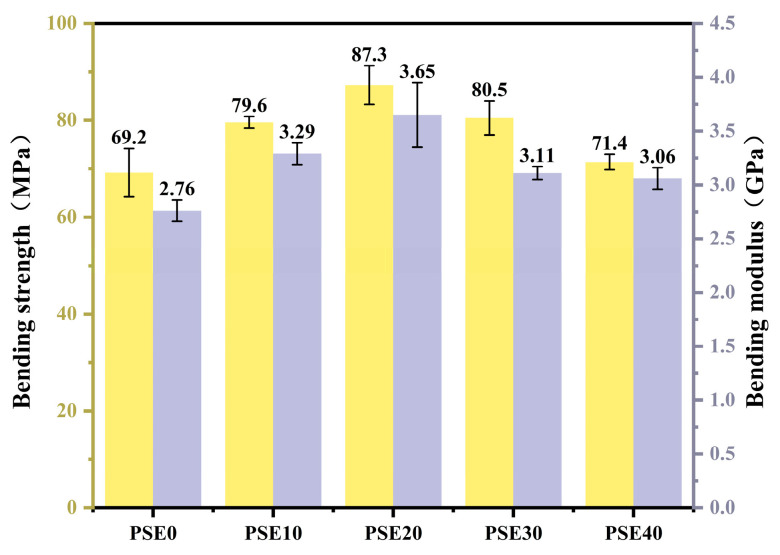
Flexural strength and flexural modulus of PSE samples.

**Figure 17:**
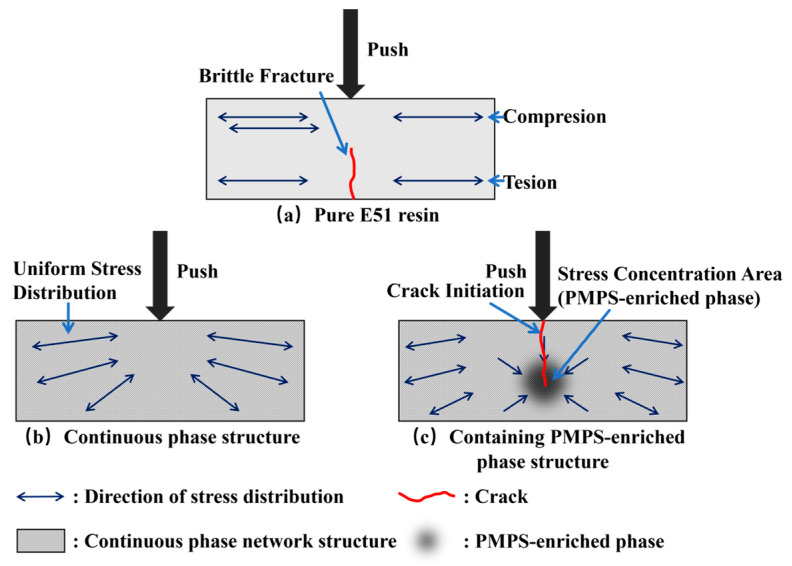
Schematic illustration of stress distribution during flexural fracture in samples containing PMPS-rich domains.

Figure 16 illustrates the variations in flexural strength and flexural modulus of the PSE systems with changing PMPS content. The neat epoxy resin exhibited a flexural strength of 69.2 MPa and a flexural modulus of 2.76 GPa. Both the flexural strength and flexural modulus exhibited a similar trend of initially increasing and then decreasing, reaching maximum values at PSE20 (87.3 MPa and 3.65 GPa, respectively), corresponding to improvements of 26.16% and 32.25% compared with the neat resin. Flexural loading is particularly sensitive to the continuity of the internal phase structure and the integrity of phase interfaces. Combined with the AFM phase images, the systems with relatively low PMPS contents exhibited uniformly distributed and interconnected network morphologies, tending to form fine-scale co-continuous structures. As illustrated in Figure 17, the co-continuous phase structure improved the load-bearing capability of the resin matrix during flexural deformation and enabled more uniform stress distribution, thereby reducing stress mismatch between the tensile and compressive regions and enhancing both flexural strength and modulus. With further increases in PMPS content, PMPS-rich domains gradually appeared, leading to coarsening of the phase domains and a reduction in interfacial continuity, which disrupted the co-continuous structure. Under flexural stress, these PMPS-rich regions readily induced stress concentration and crack initiation, resulting in simultaneous decreases in flexural strength and modulus.

#### 3.5.4. Compression Properties, Impact Toughness, and Morphological Analysis of PSE Resins

**Figure 18:**
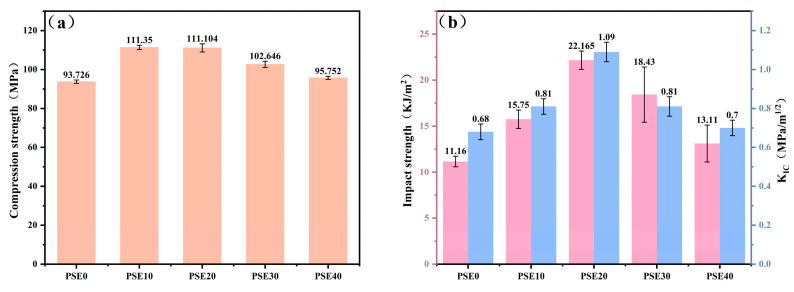
Mechanical properties of PSE systems: (**a**) Compressive strength. (**b**) Impact strength and fracture toughness.

Figure 18 presents the compressive and impact toughness of the PSE systems. As the PMPS content increased, the compressive strength first increased from 93.73 MPa for PSE0 to 111.35 MPa for PSE10 and 111.10 MPa for PSE20, representing increases of 18.80% and 18.53%, respectively, compared with the neat resin, and then gradually decreased at higher PMPS contents. A similar trend was observed for the impact strength, which increased from 11.16 kJ/m^2^ for PSE0 to 22.17 kJ/m^2^ for PSE20, corresponding to an enhancement of 98.66%, before decreasing at higher PMPS contents. To further verify the improvement in toughness, K_IC_ measurements were conducted. The results showed that, at a PMPS content of 20 wt%, the fracture toughness increased from 0.68 MPa·m^1/2^ for the neat resin to 1.09 MPa·m^1/2^, corresponding to an enhancement of 60.29%, which was consistent with the variation trend of impact strength.

At low PMPS contents (PSE10–PSE20), similar to the tensile and flexural test results, both strength and toughness were simultaneously enhanced. In this stage, PMPS improved stress transfer capability and promoted crack deflection by constructing a uniform phase structure. Meanwhile, the flexible chain segments induced local plastic deformation and energy dissipation, significantly improving toughness while maintaining strength. At high PMPS contents (PSE30–PSE40), however, the excessive soft phase reduced the load-bearing capability of the network and weakened stress transfer efficiency, thereby decreasing resistance to crack propagation.

Based on the comprehensive results of tensile, flexural, compressive, and impact tests, the PSE system containing 20 wt% PMPS exhibited the best overall mechanical performance. To further clarify the microstructural origin of the enhanced mechanical properties, the impact fracture morphology of the PSE20 sample was investigated.

**Figure 19:**
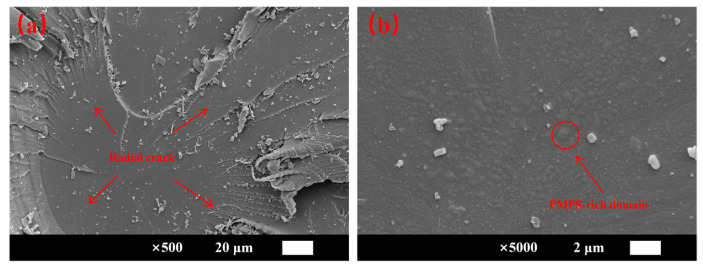
SEM images of impact fracture surfaces of PSE20: (**a**) ×500; (**b**) ×5000.

Figure 19a shows the fracture morphology at low magnification. The crack propagation exhibited radial patterns accompanied by parabolic markings, indicating repeated crack deflection and branching during crack propagation. Such tortuous crack paths increased the fracture surface area and enhanced energy dissipation. In addition, tear ridges and local step-like morphologies further indicated the occurrence of localized plastic deformation during impact fracture. At higher magnification (Figure 19b), the fracture surface appeared relatively smooth and contained uniformly distributed fine PMPS-rich domains without obvious phase boundaries. These observations suggest a relatively homogeneous distribution of PMPS within the epoxy matrix. Such a microstructure is favorable for efficient stress transfer and suppression of premature interfacial failure, thereby enabling the optimal balance among strength, stiffness, and toughness at this composition.

### 3.6. Thermal Properties of PSE Resins

#### 3.6.1. Dynamic Mechanical Analysis of PSE Resins

**Figure 20:**
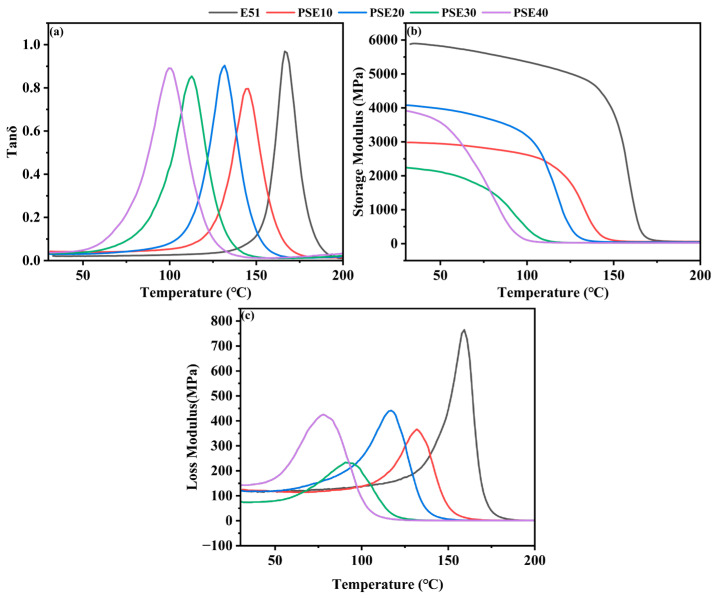
DMA curves of the PSE systems: (**a**) Loss factor (tanδ); (**b**) Storage modulus (E′); (**c**) Loss modulus (E″).

Figure 20 presents the dynamic mechanical properties of the PSE systems, and the corresponding data are summarized in Table 3. Figure 20a shows the tanδ curves, where the peak temperature corresponds to the glass transition temperature (Tg) of the material. As the PMPS content increased from 0 wt% to 40 wt%, the Tg of the modified resins decreased from 165 °C to 99 °C. This reduction can be primarily attributed to the introduction of flexible Si–O–Si segments, which enhanced chain mobility. Meanwhile, the crosslink density decreased from 4.62 × 10^3^ mol/m^3^ for PSE0 to 1.77 × 10^3^ mol/m^3^ for PSE40 with increasing PMPS content, weakening the network constraints and further promoting molecular chain motion. Figure 20c presents the loss modulus curves of the PSE systems. Similar to the tanδ results, the loss modulus peak gradually shifted toward lower temperatures with increasing PMPS content, further confirming the progressive decrease in Tg after PMPS incorporation. Although the Tg values determined from the loss modulus peaks were slightly lower than those obtained from the tanδ peaks, this difference is commonly observed in DMA because the loss modulus peak corresponds to the maximum energy dissipation, whereas the tanδ peak represents the maximum damping behavior during viscoelastic relaxation. Nevertheless, both methods exhibited the same variation trend with increasing PMPS content, confirming the reliability of the Tg determination. Furthermore, each sample exhibited only a single loss modulus peak, indicating a single dominant relaxation process and supporting the good compatibility between PMPS and the epoxy matrix. Figure 20b shows the variation in storage modulus (E′) with temperature. As the temperature increased, the storage modulus of all systems gradually decreased and underwent a rapid decline in the glass transition region. Compared with PSE0, the decrease in storage modulus for the PSE systems was more gradual, suggesting that the flexible Si–O–Si segments delayed chain relaxation and broadened the glass transition process over a wider temperature range.

According to the rubber elasticity theory [31], the crosslink density (ρ) of the cured resin was calculated using the equation ρ = E′/(3RT), where E′ is the storage modulus at Tg + 50 K, R is the gas constant (8.314 J·mol^−1^·K^−1^), and T is the absolute temperature at the corresponding measurement point. The calculated results showed that the crosslink densities of PSE0 and PSE10 remained nearly unchanged, indicating that low PMPS contents could participate in the construction of the epoxy curing network without significantly reducing the effective crosslink density. With further increases in PMPS content, both the storage modulus and crosslink density gradually decreased, suggesting that the network structure became less compact. Notably, the reductions in crosslink density and Tg did not lead to deterioration in the mechanical properties. Combined with the aforementioned phase structure and mechanical performance analyses, it can be concluded that the co-continuous phase structure induced by PMPS effectively compensated for the adverse effects associated with the reduced crosslink density, thereby contributing to the overall enhancement of the mechanical performance of the system.

#### 3.6.2. Thermogravimetric Analysis of PSE Resins

**Figure 21:**
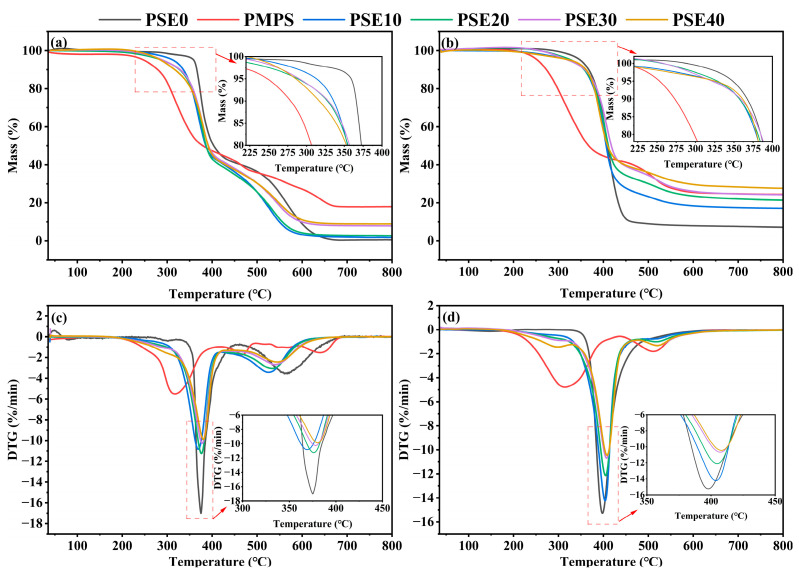
Thermal analysis curves of the PSE systems under air and nitrogen atmospheres: (**a**) TG curves in air; (**b**) TG curves in nitrogen; (**c**) DTG curves in air; (**d**) DTG curves in nitrogen.

The thermal degradation behaviors of PMPS and cured PSE systems under both air and nitrogen atmospheres were investigated by thermogravimetric analysis. The TG and DTG curves are presented in Figure 21, and the corresponding thermal degradation parameters, including the initial decomposition temperature (T_5%_), the temperature at the maximum degradation rate (T_max_), and the char yield at 800 °C, are summarized in Table 4. PMPS exhibited negligible decomposition below 200 °C. Although its T_5%_ and T_max1_ values were lower than those of the neat epoxy resin, indicating that PMPS underwent thermal degradation at a relatively earlier stage, its char yields at 800 °C were significantly higher, reaching 17.96% and 24.45% under air and nitrogen atmospheres, respectively. These results demonstrate the excellent char-forming capability of PMPS at elevated temperatures. Compared with PSE0, all PSE systems showed lower T_5%_ values. This behavior can be attributed to the terminal hydroxyl groups of PMPS, which are prone to inducing chain scission and depolymerization reactions upon heating, thereby initiating thermal decomposition at lower temperatures [32].

As shown by the DTG curves, although the Tmax1 values of the PSE systems were similar to that of PSE0, the intensity of the main degradation peak gradually decreased with increasing PMPS content, accompanied by a continuous reduction in the maximum degradation rate. This finding indicates that PMPS effectively suppressed the thermal degradation process of the system. In addition, the char yield at 800 °C increased progressively with increasing PMPS content. Among all samples, PSE40 exhibited char yields of 8.90% and 27.63% under air and nitrogen atmospheres, respectively, representing increases of 1518.2% and 284.3% compared with PSE0. This improvement is mainly attributed to the structural rearrangement of PMPS segments at elevated temperatures, which promotes the formation of a stable Si/O/C amorphous oxide protective layer. Such a protective layer effectively inhibits heat transfer and the diffusion of volatile degradation products, thereby retarding further thermal degradation and enhancing the high-temperature thermal stability of the system [33,34].

## 4. Conclusions

In this study, PMPS was synthesized from MPS and subsequently used to modify E51 epoxy resin. A series of PSE resin systems with different PMPS contents were prepared, and the evolution of their microstructures was systematically investigated to clarify the corresponding changes in mechanical properties. The mechanical performance results demonstrated that the modified resin containing 20 wt% PMPS exhibited the optimal overall performance. Compared with the unmodified resin, the tensile, flexural, compressive, and impact strengths increased by 31.26%, 26.16%, 18.53% and 98.66%, respectively. In addition, the tensile and flexural moduli increased by 38.36% and 32.25%, respectively, while the fracture toughness increased by 60.29%, achieving the synergistic enhancement of strength, stiffness, and toughness. Microstructural characterization by optical microscopy, SEM, and AFM revealed that the introduction of PMPS effectively regulated the phase structure of the epoxy matrix and promoted the formation of a co-continuous phase structure. Within this structure, the rigid epoxy phase and flexible siloxane phase were mutually interconnected, and good interfacial compatibility and interactions were observed between the two phases. Such a morphology facilitated stress transfer and energy dissipation between the phases, thereby effectively balancing stiffness and toughness and improving the overall mechanical performance of the resin system. Combined with morphology observations, epoxy value determination, and viscosity and modulus analyses, it is suggested that reactions occurred between PMPS and the epoxy resin, leading to the incorporation of PMPS into the modified epoxy system. The modulus distribution exhibited interconnected network characteristics and a gradual transition between high- and low-modulus regions. The two phases interpenetrated each other and regulated the matrix network structure through interfacial interactions, indicating a strong correlation between phase structure evolution and macroscopic mechanical properties. DMA indicated that Tg and crosslinking density gradually decreased with increasing PMPS content. TG/DTG results showed that the introduction of PMPS increased the char yield and reduced the maximum thermal degradation rate, thereby improving the thermal stability of the system to a certain extent. Unlike previously reported polysiloxane-modified epoxy systems, the high-PMPS-content resin system (PSE40) exhibited only limited phase separation characteristics while still maintaining an interconnected co-continuous structure. This result suggests that relatively strong interactions may exist between the siloxane component and the epoxy network, which helped suppress the performance deterioration caused by phase separation in conventional polysiloxane-modified epoxy systems. In previous studies, the reduction in strength of polysiloxane-modified epoxy resins was mainly attributed to phase separation and insufficient interfacial adhesion. In contrast, the PMPS-modified system developed in this work was capable of maintaining stable phase structures over a relatively wide composition range while simultaneously achieving synergistic property optimization.

It should be noted that the present study mainly focused on the phase structure at the microscale, while the nanoscale phase structure characteristics and their roles in the synergistic enhancement of strength, stiffness, and toughness remain unclear. Further investigations using higher-resolution characterization techniques are therefore needed. Owing to its excellent strengthening and toughening performance, the developed system shows promising potential for applications in structural adhesives and composite matrices, where high strength, toughness, and thermal stability are required. However, the incorporation of PMPS increases the material cost, and further optimization and evaluation are still necessary for large-scale practical applications. This strategy provides a feasible approach for regulating the microstructure and improving the comprehensive performance of epoxy resin/polysiloxane systems, thereby broadening their potential applications in composite materials.

## Figures and Tables

**Table 1:** Formulation of the cured resin systems.

Sample	E51/g	PMPS/g	MHHPA/g	DMP-30/g
PSE0	100	0	85.78	0.5
PSE10	90	10	77.20	0.5
PSE20	80	20	68.62	0.5
PSE30	70	30	60.04	0.5
PSE40	60	40	51.47	0.5

**Table 2:** Assignments of MALDI-TOF/TOF MS peaks for PMPS.

*m*/*z* [M + Na]^+^	*m*/*z* [M]	PMPS/g	n
449.7	426.7	HO[(CH_3_)(C_6_H_5_)SiO]_3_H	3
585.6	562.6	HO[(CH_3_)(C_6_H_5_)SiO]_4_H	4
721.4	698.4	HO[(CH_3_)(C_6_H_5_)SiO]_5_H	5
857.4	834.4	HO[(CH_3_)(C_6_H_5_)SiO]_6_H	6
993.4	970.4	HO[(CH_3_)(C_6_H_5_)SiO]_7_H	7

**Table 3:** DMA Data of the PSE Systems.

Sample	Tg/°C	tanδ	E′/MPa	ρ × 10^3^/(mol/m^3^)
PSE0	165	0.96	56.3	4.62
PSE10	146	0.74	53.8	4.60
PSE20	131	0.85	40.2	3.71
PSE30	112	0.80	19.7	1.90
PSE40	99	0.85	17.8	1.77

**Table 4:** Thermal Degradation Data of the PSE Systems.

Sample	Air				N_2_		
	T_5%_/°C	T_1max_/°C	T_2max_/°C	R_800°C_/%	T_5%_/°C	T_max_/°C	R_800°C_/%
PSE0	359	375	564	0.55	349	397	7.19
PMPS	248	317	467	17.96	253	314	24.45
PSE10	313	368	525	1.76	324	403	17.08
PSE20	288	376	533	2.70	328	404	21.40
PSE30	287	378	539	7.87	321	407	24.25
PSE40	285	380	544	8.90	325	408	27.63

Note: T_5%_ represents the initial thermal degradation temperature; T_1max_ and T_2max_ denote the maximum thermal degradation temperatures for the first and second stages, respectively; R_800°C_ indicates the residual char yield at 800 °C.

## Data Availability

Data is contained within the article.
